# Sir James Paget on Over-Feeding

**Published:** 1868-06

**Authors:** 


Sir James Paget on Over-feeding.—Many American physicians, catching up the fashion from a few British innovators who are bent on signalizing themselves by striking out new paths, appear to be governed by the one grand idea that disease is debility, to be overcome by cramming the stomach with beef tea and beef steaks, wine and whisky. Even the doctrine is broached that fevers are to be expelled by stimulation, and the advocates of the doctrine are not wanting in statistical proofs—for anything can be proved by statistics. Sickness produces death through starvation, we are told, and therefore alimentation is the cure. And all this is dignified with the appellation of conservative medicine! We take the liberty of thinking, in this private way, that there is a great deal of bald quackery among the dignitaries of the medical profession at the present day. The lesser lights are not slow to follow the greater, and accordingly we find not a few beardless upstarts seeking to distinguish themselves by denying all old things in medicine, and especially sneering at the old fogies of the former generation, as if children are to gain honor by proclaiming that their fathers were fools. Turning away from such mountebanks, it is pleasant to see such a man as Sir James Paget, of good old St. Bartholomew, standing by his colors, and refusing to turn summersaults for the sake of fashion or ephemeral popularity. We have been led to the above remarks by reading a lecture of Sir James, given in St. Bartholomew's Hospital, in which he takes the view that much mischief is done by over-eating, especially by the great consumption of meat and nitrogenous food. "The poor," he says, "of agricultural districts eat far less meat than those in large towns do, and are, by comparison, less fed, though probably not worse fed, and you may frequently observe that patients who come to us from agricultural districts bear operations in all respects, better than Londoners who are submitted to the same proceedings. Of course many things occur to make the differences of constitution between a town and a country population ; but I am satisfied that among these things a very potent influence is exercised by the difference of diet. And the differences that we may thus see are strongly illustrated by what one hears of the results of operations upon the natives of India and other Eastern countries, whose diet is almost exclusively vegetable. Almost any amount of injury may be inflicted on them, and not be followed by the destructive mischiefs which occur to Europeans under the same circumstances. They are defective, it is said, in healing power, but they recover with comparative certainty, however slowly, from operations of the greatest magnitude. A common expression about them is, 'You can't kill them.' "

